# Does Exercise Improve False Episodic Memory in Dementia?

**DOI:** 10.3390/jcm8111829

**Published:** 2019-11-01

**Authors:** Shigehiko Ogoh, Takeshi Hashimoto, Soichi Ando

**Affiliations:** 1 Department of Biomedical Engineering, Toyo University, 2100 Kujirai, Kawagoe-shi, Saitama 350-8585, Japan; 2 Faculty of Sport and Health Science, Ritsumeikan University, Shiga 525-8577, Japan; thashimotosci@gmail.com; 3 Graduate School of Informatics and Engineering, The University of Electro-Communications, Tokyo 182-8585, Japan; soichi.ando@gmail.com

Ageing is a risk factor involved in decline in cognitive function, particularly in executive function, processing speed and episodic memory [1,2,3]. This age-related cognitive decline is exacerbated by cardiovascular disorders and cardiovascular risk factors, such as hypertension, diabetes, obesity and neurodegenerative diseases including dementia [4,5,6]. Elderly people basically rely on episodic memory for their judgement regarding individual experience, while ageing increases both omission and commission errors. Therefore, even healthy elderly people are more susceptible to false memory than young adults [7]. False memory has more serious effects on the lifestyle of elderly people, even though it is just considered as a memory problem for young adults [8]. Patients with dementia especially exhibit high rates of memory distortions; for example, they quickly forget newly learned information [9,10]. Tat et al. [10] reported that patients with mild cognitive impairment (MCI) and Alzheimer’s disease (AD) dementia show poorer memory in both recall and recognition tests compared with that of healthy elderly people, indicating impaired episodic memory. Therefore, strategies that can reduce memory distortions in both elderly people and patients with dementia should be identified in order to improve their quality of life. Interestingly, healthy elderly controls and patients with MCI due to AD had improved memory discrimination in the item-specific condition compared with the relational condition, whereas patients with AD dementia did not, suggesting that patients with MCI due to AD use intact frontal networks to effectively engage in this strategy [10]. Furthermore, different levels of dementia may modify the episodic memory differently.

Epidemiological studies [11] demonstrated that exercise experience may decrease the incidence of dementia. Therefore, many studies have investigated the effects of exercise on cognition. However, the effects of exercise remain controversial due to widely different cognitive assessment instruments, exercise protocols (including type of exercise, length and intensity) and subject-selection criteria [12]. Landmark meta-analysis was conducted that elucidated the positive impact of aerobic exercise on the cognitive function of healthy elderly people [13]. Aerobic exercise training improves the multicognitive domain, that is, processing speed and spatial processing including memory [13]. In addition, previous studies on exercise neurobiology mainly focused on working memory capacity and retrospective recall of actual events/episodes [14]. An acute exercise has been reported to enhance retrospective episodic memory performance, and previous studies also investigated the potential underlying mechanisms of influence of acute exercise on episodic memory [15]. However, few studies have recently examined the effects of acute exercise on false memory. Green and Loprinzi [16] for the first time reported some suggestive evidence that acute exercise may reduce the rate of false memories, although an acute moderate-intensity aerobic exercise is not associated with prospective memory performance. In an excellent research manuscript [17], published in the 2018 issue of the *Journal of Clinical Medicine*, entitled “Experimental investigation of the time course effects of acute exercise on false episodic memory” by Siddiqui and Loprinzi, the authors investigated the potential time-course effect of acute exercise on false episodic memories and episodic memory function, based on their previous study [16]. They provided two important findings: First, acute exercise occurring before rather than during the memory task may be optimal in enhancing short- and long-term episodic memory function. Second, acute exercise may help reduce the rate of both short- and long-term false memories regardless of exercise duration. Recently, the same research group [18] investigated an effect of different acute exercise intensities on underlying mechanism related to true and false episodic memories. The findings of this study suggest that an acute higher-intensity exercise may enhance true episodic memories and possibly ameliorate the rate of false episodic memories. The last two previous studies [16,18] on memory function provide important information that optimal exercise enhances episodic and false episodic memory functions.

Importantly, in order to use exercise as a strategy that reduces memory distortions in both elderly people and patients with dementia, whether false episodic memory in elderly people and patients with dementia is also improved by exercise should be determined. However, these previous investigations [16,17,18] regarding false episodic memory were conducted on healthy young participants. The number of studies suggesting that physical activity and exercise have positive effects on the cognitive function of elderly people have been increasing [19]. Physical activities had a positive effect on the improvement of cognitive decline in most cases [20], and physical activity is particularly protective against AD [21]. However, it remains unclear whether physical activity and exercise directly cause brain function in patients with dementia, because the brain and cognitive functioning is likely affected by physical activity- and/or exercise-elicited physiological and psychological influences indirectly and strongly [22,23]. On the other hand, previous studies created a false memory in mice by optogenetically manipulating their memory engram-bearing cells in the hippocampus [24]. In addition, ageing and dementia cause brain atrophy in the hippocampus [25]. These findings suggest that anatomical and morphological changes in the hippocampus may be related to false episode memory function. Importantly, exercise directly affects the hippocampus and increases the size [26] and neurogenesis [27] in the hippocampus, which is associated with memory. These findings provide the possibility that exercise can improve false episodic memory due to its direct effect on the hippocampus. However, further investigations are warranted to answer these important questions.
